# Factors associated with physicians’ prescriptions for rheumatoid arthritis drugs not filled by patients

**DOI:** 10.1186/s13075-018-1580-5

**Published:** 2018-05-02

**Authors:** Hong J. Kan, Kirill Dyagilev, Peter Schulam, Suchi Saria, Hadi Kharrazi, David Bodycombe, Charles T. Molta, Jeffrey R. Curtis

**Affiliations:** 1 Center for Population Health IT, Department of Health Policy and Management, Johns Hopkins Bloomberg School of Public Health, Hampton House HH502, 624 N. Broadway, Baltimore, MD 21205 USA; 2 Cortica US, 425 Broadway, New York, NY 10013 USA; 3 Computer Science Department, Johns Hopkins University, 3400 N Charles Sreett, Baltimore, MD 21218 USA; 4 Main Line Rheumatology, Lankenau Medical Center, 100 Lancaster Avenue, Wynnewood, PA 19096 USA; 5 Division of Clinical Immunology and Rheumatology, University of Alabama at Birmingham, 510 20th Street South, Birmingham, AL 35294 USA

**Keywords:** Rheumatoid arthritis, Primary nonadherence, Disease-modifying anti-rheumatic drugs, Methotrexate, Biologics, Predictive modeling

## Abstract

**Background:**

This study estimated the extent and predictors of primary nonadherence (i.e., prescriptions made by physicians but not initiated by patients) to methotrexate and to biologics or tofacitinib in rheumatoid arthritis (RA) patients who were newly prescribed these medications.

**Methods:**

Using administrative claims linked with electronic health records (EHRs) from multiple healthcare provider organizations in the USA, RA patients who received a new prescription for methotrexate or biologics/tofacitinib were identified from EHRs. Claims data were used to ascertain filling or administration status. A logistic regression model for predicting primary nonadherence was developed and tested in training and test samples. Predictors were selected based on clinical judgment and LASSO logistic regression.

**Results:**

A total of 36.8% of patients newly prescribed methotrexate failed to initiate methotrexate within 2 months; 40.6% of patients newly prescribed biologics/tofacitinib failed to initiate within 3 months. Factors associated with methotrexate primary nonadherence included age, race, region, body mass index, count of active drug ingredients, and certain previously diagnosed and treated conditions at baseline. Factors associated with biologics/tofacitinib primary nonadherence included age, insurance, and certain previously treated conditions at baseline. The area under the receiver operating characteristic curve of the logistic regression model estimated in the training sample and applied to the independent test sample was 0.86 and 0.78 for predicting primary nonadherence to methotrexate and to biologics/tofacitinib, respectively.

**Conclusions:**

This study confirmed that failure to initiate new prescriptions for methotrexate and biologics/tofacitinib was common in RA patients. It is feasible to predict patients at high risk of primary nonadherence to methotrexate and to biologics/tofacitinib and to target such patients for early interventions to promote adherence.

## Background

Rheumatoid arthritis (RA) is a chronic autoimmune disease characterized by persistent synovitis, joint destruction, systemic inflammation, and immunological abnormalities. Available drug treatments for RA include nonsteroidal anti-inflammatory drugs (NSAIDs), glucocorticoids, and conventional disease-modifying anti-rheumatic drugs (DMARDs) such as hydroxychloroquine, leflunomide, methotrexate, and sulfasalazine. In addition, the last two decades heralded the arrival of biologic DMARDs including anti-tumor necrosis factor (anti-TNF) biologics such as adalimumab, certolizumab, etanercept, golimumab, and infliximab; and nonanti-TNF biologics such as abatacept, anakinra, rituximab, and tocilizumab, as well as newer synthetic DMARDs such as tofacitinib. Even though rheumatologists have many choices of medications to use for RA patients, patient adherence to RA medications is suboptimal [1–5], which has been one of the causes of suboptimal control of RA disease [6, 7]. It is useful to distinguish two major types of nonadherence. Primary nonadherence occurs when patients do not fill a new (first) prescription written by their physicians; secondary nonadherence occurs when patients fill a new prescription one or more times but subsequently discontinue the treatment.

Secondary nonadherence to RA treatments has been studied extensively in terms of rates and relevant factors [1–5]. For example, systematic literature reviews found that rates of methotrexate persistence ranged widely from 50 to 94% at 1 year and from 25 to 79% at 5 years [1], and that persistence to biologic DMARDs ranged from 32 to 91% at 1 year [3]. While many factors may impact adherence, a review of factors for immune-mediated inflammatory diseases including RA found consistent associations with adherence for psychosocial factors, with the strongest evidence for the impact of the healthcare professional–patient relationship, perceptions of treatment concerns and depression, lower treatment self-efficacy and necessity beliefs, and practical barriers to treatment [4]. Specifically, another systematic review found that adherence to methotrexate was mostly strongly related to beliefs in the necessity and efficacy of methotrexate, absence of low mood, mild disease, and monotherapy [5]. More generally, the importance of patient beliefs on adherence including necessity for and concerns about treatments were confirmed as important factors for adherence among many other factors across chronic diseases [8, 9]. Drug-specific characteristics and concerns may motivate adherence. For example, methotrexate may cause adverse events such as fatigue, gastrointestinal symptoms including nausea and diarrhea, malaise, oral ulcers, and alopecia [10]. A variety of other AEs specific to RA biologics may also lead to discontinuation including common and serious infections, laboratory abnormalities, and gastrointestinal perforation [11].

In contrast to many published studies on secondary nonadherence in RA where patients have experience with the medication, failure to fill a new prescription for RA medications has been understudied. In particular, factors that influence not filling a new RA prescription are not well understood. In order to conduct such research, one must link physicians’ prescriptions as written or ordered to patients’ filling of those prescriptions. Among the few studies available, Yelin et al. [12], using a longitudinal patient survey, studied sociodemographic, disease, health system, and contextual factors affecting failure to initiate biologics for RA, and found that age, Hispanic ethnicity, being married, and rural residence were associated with a higher probability of initiating biologics. Fewer rheumatology visits and living in an area with at least one federally qualified health center were associated with a lower probability of biologic initiation despite being prescribed the therapy. Harnett et al. [13], using electronic health records (EHRs) linked with claims, found that more than 50% of patients with RA who were prescribed injectable biologic DMARDs did not fill or receive administration within 30 days of the index prescription and that more than 40% of patients did not initiate treatment within 180 days. However, their study was limited to biologic DMARDs, and did not attempt to identify important predictors associated with primary nonadherence.

This population-based study of RA patients from multiple healthcare provider organizations in the USA estimated the extent of primary nonadherence to methotrexate (MTX), the most commonly prescribed conventional DMARD, and to biologic DMARDs or tofacitinib (B/T). The analysis focused on patients who were newly prescribed these medications, harnessing the power of an integrated EHRs and claims database that juxtaposes what a clinician prescribed based on EHRs and what that patient actually filled or received based on administrative claims. In addition, the study aimed to identify important predictors associated with primary nonadherence and to develop simple, transparent prediction models in order to identify RA patients at risk of primary nonadherence at the time that the prescription is written and target them for appropriate clinical interventions.

## Methods

### Sample selection

This study was designed as a retrospective cohort study of RA patients identified from Optum’s de-identified Integrated Claims-Clinical dataset, which combines adjudicated administrative claims with Humedica’s EHRs. The longitudinal clinical repository from Humedica is derived from 50+ healthcare provider organizations in the USA that include over 600 hospitals and 6500 clinics and treat more than 63 million patients. The integrated dataset includes historical administrative claim data from pharmacy claims, physician claims, and facility claims, linked with EHRs including medications prescribed and administered, laboratory results, vital signs, body measurements, diagnoses, and procedures. Clinical information from EHRs was derived from both structured EHR data fields (e.g., biometric and clinical observations) and unstructured free text using natural language processing (NLP) (e.g., signs, diseases and symptoms). The data extracted from free text using NLP rather than free text itself were made available for this study. The integrated dataset was statistically de-identified under the Expert Determination method consistent with the Health Insurance Portability and Accountability Act.

The integrated dataset contains linked claims and EHRs for about 120,000 RA patients from 2007 to 2015, from which a MTX cohort and a B/T cohort were independently extracted. The MTX cohort consists of patients newly prescribed oral or injectable methotrexate. The B/T cohort consists of patients newly prescribed a biologic DMARD or tofacitinib. The biologics and tofacitinib in the B/T cohort were treated as a single group of drugs in this study to focus on whether a patient initiated treatment after receiving a biologic or tofacitinib prescription or infusion order.

For inclusion in this analysis, patients were first required to have ≥ 2 RA diagnoses (International Classification of Diseases, Ninth Revision, Clinical Modification (ICD-9-CM): 714.0, 714.2, or 714.81) made by a physician (excluding laboratory, X-ray, and other provisional diagnoses) at least 7 days apart and within 12 months from inpatient and outpatient claims data. Subsequently, from EHRs, the first written prescription of MTX or the first prescription or infusion order for B/T after the first RA diagnosis was identified and defined as the index prescription. We conducted the search for the first RA diagnosis from claims and the first drug prescription from EHRs after diagnosis starting from 2007 onward in order to find patients at the earliest stage of disease possible allowed by the data. In addition, the following inclusion and exclusion criteria were applied. Patients must have been ≥ 18 years old at the time of the first RA diagnosis. Patients must have ≥ 12 months of continuous pharmacy and medical insurance coverage before and ≥ 2 (for MTX) or ≥ 3 (for B/T) months of continuous coverage after index prescription. Patients in the MTX cohort who ever had previous evidence of use of MTX, biologics, or tofacitinib in claims data using all available data before the index date were excluded. Patients in the B/T cohort who ever had evidence of use of biologics or tofacitinib in claims data using all available data before the index date were excluded. Also excluded were patients with diagnoses of other autoimmune diseases in claims data before the index, including psoriasis or psoriatic arthritis, inflammatory bowel disease, and ankylosing spondylitis or cancer (excluding nonmelanoma skin cancer). Patients with RA-related inpatient claims within 2 (for MTX) or 3 (for B/T) months after the index were also excluded as they may have received treatment in the hospital setting. This cohort definition is supported by previous research demonstrating that the positive predictive value of identifying RA patients exceeds 85% [14]. The 12-month period before the index prescription was defined as the baseline period during which potential predictors associated with primary nonadherence were identified from claims or EHRs.

The primary nonadherence concept was predicated on linking a new written prescription or infusion order in the EHR data to its filling/administration status in pharmacy claims or medical claims (for infused RA therapies). This study defined a new prescription as the very first prescription or infusion order of MTX or B/T after RA diagnosis. To find out how successfully these sample selection criteria identified new prescriptions, we calculated the percentage of patients with the first RA diagnosis in claims occurring at or after the first ever recorded EHR activity. This criterion guaranteed overlap of EHRs with claims data after RA diagnosis. Thus, the calculated percentage also indicates the percentage of index prescriptions being the first prescription ever recorded in the EHR data after RA diagnosis according to our definition of new prescriptions. This is a conservative measure (i.e., actual percentage of index prescriptions being the first prescription could be higher) as a patient could have been registered in an EHR system before the first recorded EHR activity occurred.

### Measures and outcomes

Primary nonadherence was defined as a new prescription of MTX or a new prescription or infusion order of B/T written by a physician as recorded in EHRs but not filled or administered within 2 months for MTX or 3 months for B/T based on claims. Since a drug filled/administered in claims could not be directly linked to a specific prescription in EHRs as administrative claims and EHR prescriptions/orders belong to two unlinked data systems, we searched for any MTX and any B/T filled/administered in claims after the index prescription and attributed the first such claim to the index prescription. Thus, we gave “credit” for any filled or administered B/T therapy, despite the fact that formulary restrictions might preclude use of certain targeted therapies and might require a different therapy to be used first. We compared the individual index B/T prescription with the first filled/administered B/T after the index, expecting that they would agree in most cases. More time was allowed for B/T initiation than for MTX as patients often need more time to clear clinical screening and insurance hurdles (e.g., screening for latent tuberculosis, prior authorization) and may face practical barriers such as scheduling and travel arrangement for infusions [15]. The time window of 2 and 3 months in the definition of primary nonadherence to MTX and B/T was also confirmed empirically by examining the distribution of time elapsed between a new prescription and the first filled or administered MTX and B/T. Infused and some self-injected biologics were identified in medical claims through the Healthcare Common Procedure Coding System, and all oral drugs and the majority of self-injected biologics were identified in pharmacy claims through National Drug Codes (NDCs).

The following baseline potential predictors for primary nonadherence were identified from claims or EHRs either at the index date or during 12 months before the index date. Using clinical judgment and subject matter expertise of the rheumatologist authors, 398 and 416 potential predictors at baseline were identified for the MTX and B/T cohorts, respectively. Baseline patient characteristics (30 variables for both the MTX and B/T samples) included age, sex, average household income (imputed values at the level of zip codes), percentage with college education (imputed values at the level of zip codes), geographic divisions, integrated delivery system (vs multispecialty practice), commercial insurance (vs Medicare), insurance products (e.g., health maintenance organization, point of service, preferred provider organization, exclusive provider organization), administrative service only, and a consumer-driven health plan which is typically associated with high out-of-pocket expenses [16]. Clinical and drug-related characteristics at baseline (17 variables for MTX and 19 variables for B/T) included the count of all active drug ingredients (not just RA related), count of chronic conditions, count of inpatient hospitalizations, count of emergency visits (all of the counts were derived from claims), number of days from RA diagnosis (derived from claims) to index prescription (derived from EHRs), number of RA diagnoses recorded in claims, calendar year of index prescription (from EHRs), prescription of both MTX and B/T within 2 months prior to index (from EHRs), and route of administration of index B/T prescriptions (namely, infusion, subcutaneous injection, or oral).

From structured EHR data fields, clinical observations (16 variables for both MTX and B/T) such as body mass index (BMI), smoking status (namely, never smoked, current smoker, not smoking now, previously smoking), pain score on a 0–10 scale, pulse, and respiration rate were included. When multiple clinical observations were available, the one closest to the index was chosen. A separate indicator for missing data was added for each clinical observation. From the NLP data extracted from free text in EHRs, we generated binary indicators (nine variables for both MTX and B/T) for the presence or absence of signs, diseases, and symptoms (pain, RA, swelling, tenderness, anxiety, depression, fatigue, weakness, and arthritis) as their mentions in free clinical text may serve as a dichotomous indicator for severity. No other measures of RA disease activity or severity were found from the current version of NLP data. Note that the NLP data extracted from free text were generated by Optum for general research purposes rather than specifically for this study or for RA.

In addition, the Johns Hopkins Adjusted Clinical Groups® (ACG®) risk adjustment/case mix system version 11.0 [17] was applied to claims data to generate general comorbidity indicators based on ICD-9-CM diagnosis codes from inpatient and outpatient claims and NDCs from pharmacy claims. These included diagnosis-based conditions called Expanded Diagnosis Clusters (EDCs; 253 variables for MTX and 234 for B/T), pharmacy-based conditions called Rx-defined Morbidity Groups (RxMGs; 59 variables for MTX and 58 for B/T), and Aggregated Diagnosis Groups (ADGs; 32 variables for both MTX and B/T). EDCs and RxMGs cover a large aggregate set of comorbidities including depression, anxiety, and sleep disorder. RxMGs represent treated conditions and do not completely overlap with EDCs which are based solely on diagnosis codes. ADGs are an even higher level aggregation of diagnosed conditions based on clinical criteria such as time limited or not, requiring primary or specialty care, or addressing physical health or psychosocial needs, as well as expected need for healthcare resources.

### Statistical analysis

Patient characteristics at baseline were summarized using mean, median, standard deviation (SD), and percentage. A chi-square test was applied to test association between two categorical variables when appropriate.

Entering all of the 400 or so potential predictors selected based on clinical judgment into a predictive model for primary nonadherence may result in overfitting given the relatively small sample size as well as inclusion of potentially irrelevant variables. To address the high-dimensional input data, the potential predictors were tested for their importance in predicting primary nonadherence using L1-penalized LASSO (least absolute shrinkage and selection operator) logistic regression. LASSO logistic regression conducts variable selection and model estimation at the same time [18]. Specifically, each cohort was first randomly divided into training (75%) and test (25%) samples. In the training sample, a 10-fold crossvalidated misclassification error was evaluated across a wide range of the L1-penalty parameter to determine the largest such penalty that was within one standard error of the parameter that achieved the minimum error. The one standard error criterion tends to select a more parsimonious model without compromising much predictive power. This is especially important for this study as we aimed to develop an interpretable, transparent model as a simple tool for clinical application to identify patients at risk of primary nonadherence. To obtain unbiased estimates of the variables selected by the LASSO logistic regression, a regular multivariate logistic regression model was estimated using the entire training sample. Age, sex, and race were forced into the final regression models regardless of variable selection by LASSO regression.

To assess generalizability of model estimates, the logistic regression estimated with the training sample was applied to the test sample. The area under the curve (AUC) of the receiver operating characteristic and the Hosmer–Lemeshow goodness-of-fit test were estimated in the test sample. To visually show the calibration of the model in the test sample, mean observed probability was plotted against mean predicted probability in each of the 10 deciles of predicted probabilities. All programming was performed in R version 3.2.2 [19] with the two key packages dplyr version 0.4.3 [20] and glmnet version 2.0–5 [21].

## Results

The MTX and B/T cohorts consisted of 763 and 434 patients (Table 1). Requiring ≥ 1 prescriptions in EHRs, continuous medical and pharmacy benefits, and no prior evidence of MTX and B/T use resulted in the largest loss of patients. In almost all of the patients (753 out of the 763 MTX patients; 430 out of the 434 B/T patients), the first identified RA diagnosis occurred at or after the first recorded EHR activity, indicating that the index prescription was indeed the very first prescription of corresponding drugs after RA diagnosis in almost all cases.

**Table 1:** Patient selection for methotrexate (MTX) and biologics/tofacitinib (B/T) cohorts

Inclusion/exclusion criteria	Number of patients for MTX cohort	Number of patients for B/T cohort
Patients with two RA inpatient/outpatient diagnoses 7 days apart and within 12 months of each other in claims; age ≥ 18 years at first such RA diagnosis	49,606	49,606
Requiring ≥ 1 prescriptions of corresponding drugs from EHR at or after first RA diagnosis (first prescription defined as index prescription)	8025	5129
Requiring ≥ 12 months before index and ≥ 2 (for MTX) or ≥ 3 (for B/T) months after index of continuous medical and pharmacy benefits coverage (≤ 45 days of insurance gap allowed)	2401	1266
Excluding patients with filling or administration of corresponding drugs in claims during 12+ months before index	968	483
Excluding patients with RA-related hospitalizations in claims within 2 (MTX) or 3 (B/T) months after index prescription	955	476
Excluding patients with B/T fills or administration in pharmacy and medical claims during 12+ months before index (i.e., biologic naïve)	848	–
Excluding patients with ≥ 2 diagnoses of psoriasis or psoriatic arthritis, inflammatory bowel disease, ankylosing spondylitis, or cancer (excluding nonmelanoma skin cancer) in claims during 12+ months before index (final sample size)	763	434

*RA* rheumatoid arthritis, *EHR* electronic health record

Among the 763 patients with a new MTX prescription, 281 failed to initiate a MTX treatment within 2 months, resulting in a MTX primary nonadherence rate of 36.8% (95% confidence interval (CI) ± 3.4%). Similarly, 176 out of the 434 patients with a new B/T prescription failed to initiate a B/T treatment within 3 months, resulting in a B/T primary nonadherence rate of 40.6% (95% CI ± 4.6%).

Table 2 presents patient characteristics at the index date or during 12 months before the index date. These characteristics (except for age, sex, and race) were selected as important variables by the LASSO logistic regression. At the index date, the mean age of B/T and MTX patients was 57.7 and 62.5 years, respectively. About 75% of the patients were female and about 80% were white in both the cohorts. More B/T patients (61.5%) had commercial insurance than MTX patients (47.7%). Although neither of them was selected as important predictors, the median percentage of patients with college or higher education (based on zip codes) was 23.0% and 23.5% for MTX and B/T patients, respectively, and the median average household income (based on zip codes) was $42,248 and $41,972 for MTX and B/T patients, respectively. In the MTX cohort, 19.5% of patients recorded healthy BMI (18.5–24.9), the mean count of active drug ingredients was 7.9, and the median number of days between RA diagnosis and index MTX was 90. Pharmacy-based previously treated musculoskeletal/inflammatory conditions (MUSx030), which include RA, were seen in 83.4% of B/T patients, indicating a high treatment rate for these conditions among B/T patients before index prescription. Note that patients previously treated with conventional DMARDs were allowed in the B/T cohort.

**Table 2:** Baseline characteristics of patients at index or during 12 months before index prescription

	Methotrexate (MTX)			Biologics/tofacitinib (B/T)		
	Noninitiators (*n* = 281)	Initiators (*n* = 482)	Total (*n* = 763)	Noninitiators (*n* = 176)	Initiators (*n* = 258)	Total (*n* = 434)
Age at index prescription, mean (SD)	67.6 (12.7)	59.5 (13.5)	62.5 (13.8)	63.0 (13.7)	54.0 (13.4)	57.7 (14.2)
Female (%)	73.3	71.6	72.2	74.4	77.1	76.4
Race (%)						
White	87.5	78.4	81.8	83.0	80.2	81.3
African American	5.7	8.1	7.2	5.1	7.4	6.5
Asian	2.1	2.1	2.1	0.6	1.2	0.9
Other/unknown	4.6	11.4	8.9	11.4	11.2	11.3
Geographic division (%)^a^						
East North Central	14.2	23.2	19.9			
East South Central	1.8	0.8	1.2			
Middle Atlantic	5.7	10.0	8.4			
Mountain	2.1	2.9	2.6			
New England	16.7	3.1	8.1			
Other/unknown	1.8	2.5	2.2			
Pacific	8.2	8.9	8.7			
South Atlantic/West South Central	24.2	36.9	32.2			
West North Central	25.3	11.6	16.6			
Commercial insurance (vs Medicare) at index (%)^a,b^	29.9	58.5	47.7	34.1	79.5	61.5
Body mass index during 12 months before index (from EHRs) (%)^a^						
Underweight (< 18.5)	1.4	2.1	1.8			
Healthy weight (18.5–24.9)	16.4	21.4	19.5			
Overweight (25.0–29.9)	20.6	23.9	22.7			
Obesity I (30.0–34.9)	11.7	18.5	16.0			
Obesity II (35.0–39.9)	6.4	8.5	7.7			
Obesity III (40.0+)	5.3	7.9	6.9			
Missing	38.1	17.8	25.3			
Active ingredient counts during 12 months before index (from claims)^a^, mean (SD)	3.4 (6.4)	10.5 (6.8)	7.9 (7.5)			
Number of days between RA diagnosis (from claims) and first prescription (from EHRs)^a^, median; mean (SD)	371	39	90			
	602	279	398			
	(698)	(502)	(602)			
Diagnosis-based comorbidity indicators (EDCs) during 12 months before index (based on claims) (%)						
ADM05: administrative concerns and nonspecific laboratory abnormalities^a^	70.5	60.0	63.8			
GUR11: incontinence^a^	6.0	1.2	3.0			
Pharmacy-based comorbidity indicators (RxMGs) during 12 months before index (based on claims) (%)						
MUSx020: musculoskeletal/inflammatory conditions^b^				71.6	91.5	83.4
ALLx030: allergy/immunology/chronic inflammatory^a,b^	14.2	66.6	47.3	47.2	76.0	64.3
CARx030: cardiovascular/high blood pressure^a^	13.5	45.4	33.7			
GSIx020: general signs and symptoms/pain^a^	17.8	59.1	43.9			
GSIx030: general signs and symptoms/pain and inflammation^a^	12.5	56.4	40.2			
INFx020: infections/acute minor^a^	18.1	61.2	45.3			
ENDx040: endocrine/diabetes without insulin^a^	0.7	11.0	7.2			

*SD* standard deviation, *EHR* electronic health record, *RA* rheumatoid arthritis, *EDC* Expanded Diagnosis Cluster, *RxMG* Rx-defined Morbidity Group

^a^Important predictors selected by LASSO logistic regression for predicting MTX primary nonadherence

^b^Important predictors selected for predicting B/T primary nonadherence; age, sex, and race were added to the predictive models regardless

Table 2 also compares baseline characteristics between patients who initiated treatment and those who did not. Compared to treatment initiators, noninitiators tended to be older, more likely to be white, less likely to have commercial insurance, and to have lower prevalence of the pharmacy-based RxMG conditions in both cohorts. Compared to MTX initiators, MTX noninitiators appeared to have a lower active drug ingredient count, longer wait time between RA diagnosis and index prescription, higher prevalence of the diagnosis-based EDC conditions, and a somewhat different geographic distribution.

Table 3 presents details of index B/T prescriptions and their filling/administration status. In most cases (240 out of 258 index prescriptions), the first B/T filled or administered after the index was the same drug as the index B/T prescription. Adalimumab and etanercept accounted for 73.7% of all index B/T prescriptions. In total, 86.9% of index B/T prescriptions were anti-TNFs, 9.9% were non-anti-TNF biologics, and 3.2% were tofacitinib; and 82.7% were subcutaneous injection, 14.1% were intravenous infusion, and 3.2% were oral. After collapsing the index prescriptions into the three types of drugs and the three routes of administration, a chi-square test revealed that the percentage of patients with primary nonadherence was not significantly different across the drug types (*p* = 0.368) or across routes of administration (*p* = 0.335).

**Table 3:** Biologics/tofacitinib index prescriptions, filling/administration status, and discrepancies between index prescription and first filling/administration post index

Index prescription drug	Total number of patients (*a* + *b*)	Number of patients with no B/T filled/administered within 3 months, *n* (%) (*a*)		Number of patients with ≥1 B/T filled/administered within 3 months, *n* (%) (*b*)		First B/T filled/administered after index that was different from the index prescription (among *b*)
Anti-TNF biologics						
Adalimumab (SC)	118	56	(47.5)	62	(52.5)	10 etanercept
Certolizumab (SC)	11	6	(54.5)	5	(45.5)	1 certolizumab IV
Etanercept (SC)	202	65	(32.2)	137	(67.8)	1 adalimumab
Golimumab (SC)	17	8	(47.1)	9	(52.9)	None
Golimumab (IV)	1	–		1	(100.0)	None
Infliximab (IV)	28	14	(50.0)	14	(50.0)	None
Non-anti-TNF biologics						
Abatacept (IV)	23	8	(34.8)	15	(65.2)	5 abatacept SC
Abatacept (SC)	9	5	(55.6)	4	(44.4)	None
Anakinra (SC)	1	–		1	(100.0)	None
Rituximab (IV)	7	4	(57.1)	3	(42.9)	1 abatacept SC
Tocilizumab (IV)	2	1	(50.0)	1	(50.0)	None
Tocilizumab (SC)	1	1	(100.0)	–		–
New synthetic DMARD						
Tofacitinib (oral)	14	8	(57.1)	6	(42.9)	None
Total	434	176	(40.6)	258	(59.4)	18 discrepancies

*B/T* biologics/tofacitinib, *TNF* tumor necrosis factor, *SC* subcutaneous, *IV* intravenous infusion, *DMARD* disease-modifying anti-rheumatic drug

Table 4 presents regular logistic regression model estimates for predicting primary nonadherence in the training samples. For MTX, older age, certain regions (e.g., New England and West North Central), having higher/missing BMI, and certain diagnosis-based EDC conditions (incontinence, administrative concerns, and nonspecific laboratory abnormality) were associated with a higher probability of MTX primary nonadherence. Being other/unknown in race compared to white was significantly associated with a higher probability of filling MTX. So was more active drug ingredients and having previously treated RxMG conditions such as allergy/immunology/chronic inflammatory, general signs and symptoms/pain and inflammation, diabetes without insulin, and cardiovascular/high blood pressure.

**Table 4:** Logistic regression for predicting primary nonadherence in the MTX and B/T training samples

	Methotrexate (MTX) (*n* = 584)			Biologics/tofacitinib (B/T) (*n* = 323)		
	Odds Ratio	2.5%	97.5%	Odds Ratio	2.5%	97.5%
Intercept	2.68	0.44	17.38	5.36**	1.70	17.81
Age at index prescription (vs 18–44 years)						
45–54 years	1.87	0.71	5.25	2.33#	0.97	5.90
55–64 years	0.87	0.32	2.46	1.48	0.60	3.76
65–69 years	2.30	0.56	9.87	2.32	0.64	8.65
70–74 years	1.90	0.44	8.26	2.87	0.81	10.65
75–79 years	2.24	0.50	10.18	1.78	0.46	6.94
80+ years	4.92*	1.03	24.81	2.45	0.55	11.50
Male (vs female)	0.69	0.35	1.35	1.37	0.73	2.58
Race (vs white)						
African American	1.20	0.44	3.18	0.50	0.17	1.37
Asian	0.21	0.02	1.44	1.66	0.07	20.14
Other/unknown	0.27*	0.09	0.73	1.20	0.47	2.95
Geographic division (vs East North Central)^a^						
East South Central	1.83	0.17	21.00			
Middle Atlantic	1.21	0.40	3.59			
Mountain	2.46	0.39	14.75			
New England	3.76#	0.89	16.55			
Other/unknown	0.90	0.14	5.46			
Pacific	1.74	0.56	5.24			
South Atlantic/West South Central	1.69	0.78	3.79			
West North Central	2.44#	0.94	6.43			
Commercial vs Medicare at index^a,b^	1.17	0.45	3.09	0.19***	0.08	0.43
Body mass index (vs healthy weight)^a^						
Underweight (< 18.5)	0.98	0.08	6.96			
Overweight (25.0–29.9)	1.03	0.44	2.42			
Obesity I (30.0–34.9)	1.08	0.43	2.67			
Obesity II (35.0–39.9)	1.11	0.34	3.41			
Obesity III (40.0+)	1.64	0.51	5.07			
Missing	3.28**	1.47	7.51			
Active drug ingredient count during 12 months before index (vs 0)^a^						
1–5	0.13***	0.04	0.37			
6–10	0.06***	0.02	0.24			
11–15	0.08**	0.02	0.36			
16+	0.06**	0.01	0.35			
Time between RA diagnosis and first prescription (years)^a^	1.13	0.95	1.34			
Diagnosis-based comorbidity indicators (EDCs) during 12 months before index						
ADM05: administrative concerns and nonspecific laboratory abnormalities^a^	2.21*	1.22	4.10			
GUI11: incontinence^a^	7.96**	1.92	35.40			
Pharmacy-based comorbidity indicators (RxMGs) during 12 months before index						
MUSx020: musculoskeletal/inflammatory conditions^b^				0.28**	0.12	0.63
ALLx030: allergy/immunology/chronic inflammatory^a,b^	0.27***	0.14	0.50	0.34***	0.19	0.60
CARx030: cardiovascular/high blood pressure^a^	0.40*	0.18	0.84			
GSIx020: general signs and symptoms/pain^a^	0.63	0.32	1.22			
GSIx030: general signs and symptoms/pain and inflammation^a^	0.40**	0.21	0.74			
INFx020: infections/acute minor^a^	0.88	0.43	1.79			
ENDx040: endocrine/diabetes without insulin^a^	0.15#	0.01	0.86			

*RA* rheumatoid arthritis, *EDC* Expanded Diagnosis Cluster, *RxMG* Rx-defined Morbidity Group

*p < 0.05

**p < 0.01

***p < 0.001

#*p* < 0.1

^a^Important predictors selected by LASSO logistic regression for predicting MTX primary nonadherence

^b^Important predictors selected for predicting B/T primary nonadherence; age, sex, and race were added to the predictive models regardless

For B/T, older age was associated with a higher probability of B/T primary nonadherence. Having commercial insurance and having previously treated RxMG conditions such as allergy/immunology/chronic inflammatory, and musculoskeletal/inflammatory were highly significantly associated with filling or administration of B/T.

**Fig. 1:**
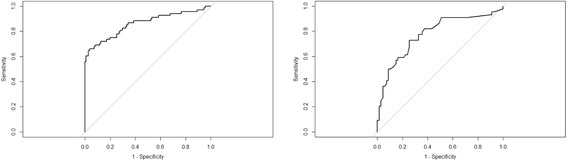
Receiver operating characteristic curve of predicting primary nonadherence in methotrexate and biologics/tofacitinib test samples. Receiver operating characteristic curve for (left) methotrexate (*n* = 179, area under the curve (AUC) = 0.86) and (right) biologics/tofacitinib (*n* = 111, AUC = 0.78)

**Fig. 2:**
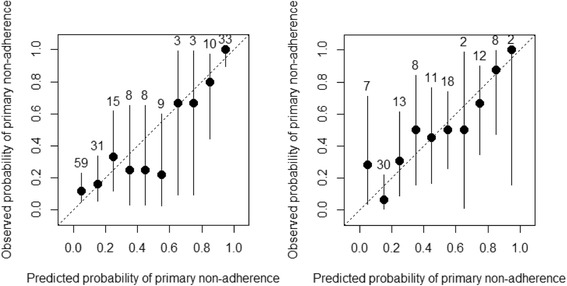
Calibration of predicted vs observed probability of primary nonadherence in methotrexate and biologics/tofacitinib test samples. Calibration plot for (left) methotrexate (*n* = 179, Hosmer–Lemeshow test *p* = 0.014) and (right) biologics/tofacitinib (*n* = 111, Hosmer–Lemeshow test *p* = 0.484). Numbers above each line of calibration plot refer to number of patients in each decile of predicted probability in the test sample. The 45° line indicates perfect calibration

The logistic regression estimated with the training samples was applied to the independent test samples. The estimated AUC was 0.86 and 0.78 for predicting MTX and B/T primary nonadherence, respectively (Fig. 1). Figure 2 shows the calibration plot of predicted vs observed probability of primary nonadherence in the MTX and B/T test samples, with the 45° line indicating perfect calibration. The Hosmer–Lemeshow test yields *p* = 0.014 for MTX primary nonadherence prediction and *p* = 0.484 for B/T, indicating acceptable calibration under α = 0.01. Visual inspection of the predicted vs observed adherence behavior suggested that the prediction model yielded reasonable accuracy, especially in identifying patients who were not adherent (high end of the *x* axis of Fig. 2).

## Discussion

This study estimated the primary nonadherence rate using integrated EHRs and claims data. It confirmed serious primary nonadherence to biologic and new synthetic DMARDs in RA patients, with 41% of patients failing to initiate a new prescription within 3 months, and the primary nonadherence rate did not differ significantly across the types of B/T drugs or routes of administration. Primary nonadherence to methotrexate was also serious, with 37% of patients failing to initiate within 2 months. This high primary nonadherence across RA drugs may not have been fully recognized by practicing physicians.

The predictive models for MTX and B/T primary nonadherence were validated in independent test samples with satisfactory discriminatory power according to the AUC (0.86 for MTX and 0.78 for B/T). Calibration of the two models was good throughout the range of predicted probabilities, which is important for real-world prediction to effectively differentiate both low and high risk. This level of predictive performance was achieved with a relatively small number of simple predictors commonly available through any EHR data sources, demonstrating the feasibility of applying the models in the routine clinical setting for identifying patients with varying levels of risk of primary nonadherence.

Older age was associated with a higher probability of primary nonadherence to both MTX and B/T. Insurance stood out as a highly significant predictor for B/T primary nonadherence. This is consistent with the finding that the higher the out-of-pocket cost, the less likely a member of a Medicare Advantage and Prescription Drug plan is to initiate a biologic DMARD therapy for RA [22] and the finding that the vast majority of Medicare prescription drug plans require sufficiently high cost sharing for biologic DMARDs to risk significant financial burden to RA patients [23]. Previous treatment experience in terms of a larger number of all active drug ingredients taken (for MTX) and certain previously treated pharmacy-based conditions (for both MTX and B/T as presented in Table 4) appeared to be associated with a higher probability of initiating the first RA prescription. Thus, it may be fruitful to target patients with a lack of previous treatment experience with the comorbid conditions identified in the model for interventions to improve primary adherence. In addition, incontinence had a large impact on MTX primary nonadherence (odds ratio = 7.96), due possibly to the fact that urinary incontinence is most prevalent and strongly associated with frailty in the elderly population [24], turning it into a proxy for frailty. In fact, the mean age of MTX patients with incontinence is 69.1 years vs 62.3 years for those without incontinence.

It is important to note that variables that were not selected as important predictors are equally as informative of primary nonadherence behavior as those selected. Overall disease burden such as the count of chronic diseases at baseline did not turn out to be an important predictor. RA-related clinical characteristics such as pain, swelling, tenderness, anxiety, depression, fatigue, and weakness did not appear to be important predictors. These binary indicators may be missing for many patients and may not capture enough granularity such as severity. However, one exception is the 0–10 pain score from clinical observations which is available to the majority (~ 65%) of patients in both the cohorts. Moderate differences in mean pain scores were observed between noninitiators (1.48 for B/T and 1.25 for MTX) and initiators (1.59 for B/T and 1.61 for MTX). In a sensitivity analysis, we added the pain score into the regular logistic regression models, and it did not turn out to be significant in either cohort. In addition, drug-related characteristics such as route of administration for B/T and combination prescription of MTX and B/T did not turn out to be important predictors. Income and education, imputed based on zip codes patients resided in, also did not appear to be important predictors.

Biologics/tofacitinib primary nonadherence was harder to predict compared to MTX as indicated by its lower AUC score, possibly because B/T risk–benefit profiles are more complex to be communicated to and understood by patients, whereas MTX risk benefit may be better understood by patients and the need for any RA treatment may be more compelling, making prior treatment experience and behavior with certain comorbid conditions more predictive of MTX primary adherence. Another possible explanation is that most patients prescribed B/T are likely to have already experienced or been prescribed MTX, and thus have already accepted the concept of disease-modifying therapy for RA, which may have impacted their beliefs and expectations for subsequent treatments and, consequently, their treatment-related behavior.

RA continues to rank as one of the costliest specialty therapy classes [25]. Although it is critical to identify appropriate patients to treat, it is equally important to proactively promote optimal primary and secondary adherence among appropriate patients at risk of nonadherence. Patients with a high predicted probability of primary nonadherence may be appropriate targets of multifaceted system, provider, and patient-level interventions to promote treatment initiation and improve treatment outcomes [26].

### Limitations

Our definition of primary nonadherence was based on identifying filling/administration of MTX or B/T in claims data. Although the current data system does not allow direct linkage of a drug filled/administered to a specific prescription, attribution can be inferred based on the temporal relationship since, in most cases, the first B/T filled/administered was the same as the index B/T prescription. We speculate that the few discrepancies observed between the index B/T prescription and the first filled/administered drug, the majority of which involved etanercept and adalimumab, could be due to insurance and other reasons.

Although we aimed to find patients in the earliest stage of RA allowable by the data, it is possible that some of them may have had a RA diagnosis before the first recorded RA diagnosis found in claims due to left-censoring in incomplete claims data. This was suggested in the B/T cohort by the small number of patients prescribed non-anti-TNF biologics or tofacitinib as the seemingly first-line therapy. These drugs typically are not used as first-line therapy after MTX failure, suggesting left-censoring. However, it is noteworthy that the mean age at first RA diagnosis in our sample is not drastically different from that of incident cohorts from other claims-based studies [27]. Despite the fact that all of the RA patients may not have been incident cases, index prescriptions were “incident” in the sense of virtually all of them being new prescriptions after RA diagnosis and no filling or administration of corresponding drugs recorded in claims for at least 12 months prior to index prescription. This study focused on filling and administration of new prescriptions rather than new prescriptions among strictly defined incident RA cases.

Another limitatoin is that filling a drug does not necessarily mean taking the drug by the patient. Note that this, however, is not an issue for infused biologics since actual administration was recorded in medical claims. Nevertheless, it is interesting to study whether a prescription was even filled and picked up by the patient since this is a necessary action to take the medication. Since this study focused on patient adherence behavior, we tried to identify as many patient-reported variables as possible. However, formal instruments for patient-reported outcomes such as the Health Assessment Questionnaire and RAPID3 were not available for a large enough sample through the current NLP processed free text data. We believe that patient-reported factors including their beliefs and perceptions of treatments are important for understanding primary adherence behavior and worth more research in the future.

Finally, the automatic variable selection procedure implemented via LASSO logistic regression cannot necessarily recover the true set of underlying parameters for an outcome. We used clinical judgment in selecting the initial pool of potential predictors and included demographic variables such as age, sex, and race regardless of the LASSO regression results. Although the emphasis of our regression model is more on prediction than explanation [28], the final selected variables generally do seem to make clinical sense. Some of them may be proxies for other unmeasured factors or latent constructs (e.g., incontinence as a proxy for frailty). The final set of predictors was also validated in a separate independent test sample. The relationship found between the predictors and outcome can only be interpreted as associative rather than causal.

## Conclusions

This study confirmed serious primary nonadherence not only to biologics/tofacitinib but also to methotrexate. With a small number of simple predictors including age, sex, race, BMI, insurance, region, prior drug count, and certain previously diagnosed and treated conditions, it is feasible to predict patients with high risk of primary nonadherence to biologics/tofacitinib and to methotrexate. Models developed in this study are potentially useful for providers to identify patients at high risk of primary nonadherence when prescribing a new RA medication in clinical care and allow the implementation of targeted interventions to improve drug initiation. How much improved primary adherence may result in improved patient outcomes and cost savings is an interesting topic for future research.

## Abbreviations

ACG®: Johns Hopkins Adjusted Clinical Groups®
ADG: Aggregated Diagnosis Group
AUC: Area under the curve of receiver operating characteristic
B/T: Biologic DMARDs or tofacitinib
BMI: Body mass index
CI: Confidence interval
DMARD: Disease-modifying anti-rheumatic drug
EDC: Expanded Diagnosis Cluster
EHR: Electronic health record
ICD-9-CM: International Classification of Diseases, Ninth Revision, Clinical Modification
MTX: Methotrexate
NDC: National Drug Code
NLP: Natural language processing
NSAID: Nonsteroidal anti-inflammatory drug
RA: Rheumatoid arthritis
RxMG: Rx-defined Morbidity Groups
SD: Standard deviation
TNF: Tumor necrosis factor
